# Long-term PM_2.5_ Exposure and Neurological Hospital Admissions in the Northeastern United States

**DOI:** 10.1289/ehp.1408973

**Published:** 2015-05-15

**Authors:** Marianthi-Anna Kioumourtzoglou, Joel D. Schwartz, Marc G. Weisskopf, Steven J. Melly, Yun Wang, Francesca Dominici, Antonella Zanobetti

**Affiliations:** 1 Department of Environmental Health,; 2 Department of Epidemiology, and; 3 Department of Biostatistics, Harvard T.H. Chan School of Public Health, Boston, Massachusetts, USA

## Abstract

**Background:**

Long-term exposure to fine particles (particulate matter ≤ 2.5 μm; PM_2.5_) has been consistently linked to heart and lung disease. Recently, there has been increased interest in examining the effects of air pollution on the nervous system, with evidence showing potentially harmful effects on neurodegeneration.

**Objective:**

Our objective was to assess the potential impact of long-term PM_2.5_ exposure on event time, defined as time to first admission for dementia, Alzheimer’s (AD), or Parkinson’s (PD) diseases in an elderly population across the northeastern United States.

**Methods:**

We estimated the effects of PM_2.5_ on first hospital admission for dementia, AD, and PD among all Medicare enrollees ≥ 65 years in 50 northeastern U.S. cities (1999–2010). For each outcome, we first ran a Cox proportional hazards model for each city, adjusting for prior cardiopulmonary-related hospitalizations and year, and stratified by follow-up time, age, sex, and race. We then pooled the city-specific estimates by employing a random effects meta-regression.

**Results:**

We followed approximately 9.8 million subjects and observed significant associations of long-term PM_2.5_ city-wide exposure with all three outcomes. Specifically, we estimated a hazard ratio (HR) of 1.08 (95% CI: 1.05, 1.11) for dementia, an HR of 1.15 (95% CI: 1.11, 1.19) for AD, and an HR of 1.08 (95% CI: 1.04, 1.12) for PD admissions per 1-μg/m^3^ increase in annual PM_2.5_ concentrations.

**Conclusions:**

To our knowledge, this is the first study to examine the relationship between long-term exposure to PM_2.5_ and time to first hospitalization for common neurodegenerative diseases. We found strong evidence of association for all three outcomes. Our findings provide the basis for further studies, as the implications of such exposures could be crucial to public health.

**Citation:**

Kioumourtzoglou MA, Schwartz JD, Weisskopf MG, Melly SJ, Wang Y, Dominici F, Zanobetti A. 2016. Long-term PM_2.5_ exposure and neurological hospital admissions in the northeastern United States. Environ Health Perspect 124:23–29; http://dx.doi.org/10.1289/ehp.1408973

## Introduction

Long-term exposure to PM_2.5_, particles with aerodynamic diameter ≤ 2.5 μm, has been consistently associated with a series of outcomes including but not limited to mortality (Krewski et al. 2009), cardiovascular (Puett et al. 2009), and cerebrovascular (Stafoggia et al. 2014) events, and lung cancer (Hamra et al. 2014).

Recently, there has been increased interest in the effects of air pollution on the central nervous system (CNS) and neurodegeneration. Particle exposure has been associated with decreased cognitive function (Power et al. 2011), accelerated cognitive decline (Weuve et al. 2012), and Parkinson’s disease (PD) hospitalizations (Zanobetti et al. 2014). Toxicological studies have provided further evidence of an association between particulate air pollution and neurodegeneration, highlighting potential biological pathways such as systemic inflammation (Block et al. 2007, 2012), which has also been consistently linked with particle exposure (Madrigano et al. 2010; Rückerl et al. 2006). Based on their findings on the effects of air pollution on altered brain innate immune response and on neuroinflammation in particular, Calderón-Garcidueñas et al. (2008b) urged that air pollution be considered a risk factor for both Alzheimer’s disease (AD) and PD.

AD and PD are the two most prevalent neurodegenerative diseases (Maragakis and Rothstein 2006). AD is the most common form of dementia (Blennow et al. 2006); in 2013, an estimated 5.2 million Americans had AD, and between 1999 and 2010, the proportion of deaths resulting from AD in the United States increased by 68% (Alzheimer’s Association 2013). PD is the most common serious movement disorder in the world (Samii et al. 2004), with an estimated age- and sex-adjusted incidence rate of 13.4 per 100,000 person years (Van Den Eeden et al. 2003). Tschanz et al. (2011) reported that the progression of disease is slow for a significant proportion of patients with neurodegenerative diseases, and for AD specifically, and urged the identification of modifiable factors that may further slow neurodegenerative progression.

The association between long-term exposure to ambient air pollution and PD/AD has not been explored in large-scale epidemiologic studies, with the exception of three studies that examined the relationship between airborne metal exposures and PD and showed evidence suggestive of the harmful effects of manganese (Finkelstein and Jerrett 2007; Willis et al. 2010) and mercury (Palacios et al. 2014). Moreover, although there is some evidence that air pollution may be involved in the initiation of neurodegeneration (Calderón-Garcidueñas et al. 2008a, 2013), we propose that it might also be involved in disease progression, potentially by worsening intermediate processes such as oxidative stress, systemic inflammation, and neuroinflammation, and by accelerating, through these pathways, the occurrence of first hospital admission. Holmes et al. (2009), for instance, reported that both acute and chronic systemic inflammation are associated with an increase in cognitive decline among early AD patients.

In this study, we investigated the effects of long-term exposure to PM_2.5_ on event time, defined as time of first hospital admission for PD, AD, or dementia in an elderly population across the northeastern United States. Specifically, we investigated whether city-wide PM_2.5_ exposure was associated with accelerated disease progression, leading to the first hospital admission. To do so, we used data from approximately 9.8 million Medicare enrollees residing in 50 cities in the northeastern United States between 1999 and 2010. We used a recently published statistical approach (Kioumourtzoglou et al. 2015) that had previously been used to assess whether yearly fluctuations in PM_2.5_ concentrations were associated with yearly fluctuations in mortality. In the present study, we applied the same approach to assess associations with yearly fluctuations in the time of first hospitalization for each of the three outcomes of interest. Our proposed approach effectively randomized exposures with respect to the most plausible covariates by eliminating potential confounding by long-term trends and by factors that vary across cities.

## Methods

**Figure 1:**
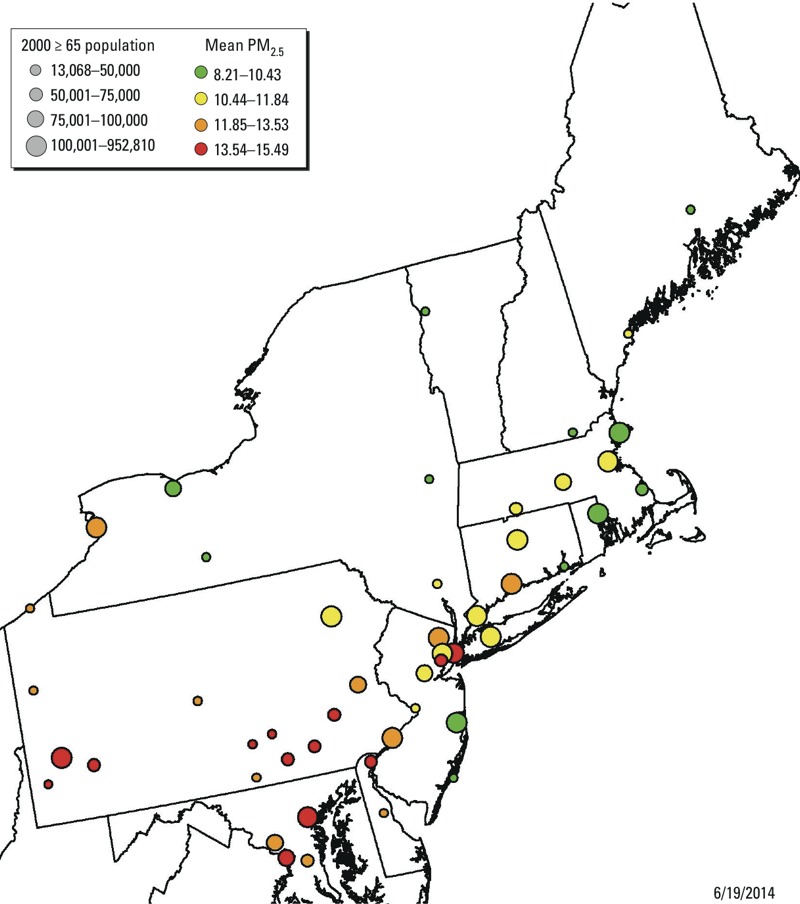
Map of the 50 cities included in our analyses. The size of the circles represents the size of the population ≥ 65 years living in each city (U.S. Census Bureau 2000) and the color indicates the average PM_2.5_ concentrations (μg/m^3^).

*Data collection.* Study population. Data were obtained from approximately 9.8 million fee-for-service Medicare enrollees (≥ 65 years old) from 50 cities across the northeastern United States, specifically from cities in Connecticut (CT), Delaware (DE), Maine (ME), Maryland (MD), Massachusetts (MA), New Hampshire (NH), New Jersey (NJ), New York (NY), Pennsylvania (PA), Rhode Island (RI), and Vermont (VT), and from Washington, D.C. (DC) for the years 1999–2010. Enrollment records were obtained from the Center for Medicaid and Medicare (CMS) (Dominici et al. 2006; Greven et al. 2011; Zeger et al. 2008). These states and cities were chosen because of data availability and because researchers have observed higher effect estimates of PM_2.5_ in the Northeast than in other U.S. regions for outcomes such as mortality (Zanobetti and Schwartz 2009; Zeger et al. 2008) and cardiovascular mortality (Puett et al. 2009). A map showing the locations of the 50 cities included in our analyses is presented in Figure 1. This study was conducted under a protocol approved by the Harvard T.H. Chan School of Public Health Human Subjects Committee.

Medicare is an open cohort; subjects entered our cohort in 1999, or upon their enrollment after 1999 (when they turned 65). For each enrollee, a record was created for each year of follow-up, which started on 1 January following entry into the cohort, and each subject was followed over time until the event (first admission for any of the outcomes of interest), or until the year of his or her death or the end of our study period (December 2010).

We also obtained the date of and primary and secondary diagnoses for each admission, which were linked to the annual records using the unique IDs of each enrollee. Specifically, using codes from the *International Classification of Diseases, Ninth Revision, Clinical Modification* (ICD-9-CM), we obtained admission records for PD (code 332), AD (code 331.0), dementia (code 290), congestive heart failure (CHF; code 428), myocardial infarction (MI; code 410), chronic obstructive pulmonary disease (COPD; codes 490–492, 494–496), and diabetes (code 250), as well as the severity of each admission, expressed as the number of days spent in the coronary or intensive care unit.

Individual-level information was available for cause-specific admissions, sex, age, race, and ZIP code of residence. Information on individual-level risk factors, such as individual socioeconomic status (SES), smoking, and diet, is not available for Medicare enrollees. We used ZIP code–level median income obtained from the 2000 U.S. Census Bureau (2000) as a proxy for SES.

Air pollution data. We obtained PM_2.5_ data from the U.S. Environmental Protection Agency’s (EPA) Air Quality System (AQS) database (U.S. EPA 2013). We estimated annual PM_2.5_ averages within each city for the period of 1999–2010. If multiple monitors were available in a city, we used the average of all monitors. Within cities and for each follow-up year, each participant was assigned annual (1 January–31 December) city-average PM_2.5_ mass concentrations as a time-varying exposure.

*Data analysis.* Health models. We ran separate models for each outcome of interest, that is, PD, AD, and dementia, using the first available, either primary or secondary, hospitalization for these conditions. We fit time-varying Cox proportional hazards models separately for each city. City-wide annual PM_2.5_ concentrations were included as the time-varying exposure of interest, as well as a term for calendar year (linear). We employed the counting process extension of the model by Andersen and Gill (1982) to create multiple observations per subject, with each observation representing a single person-year of follow-up.

We fit city-specific models to avoid confounding by factors that varied across cities. By also adjusting for calendar year, we estimated whether year-to-year variations in PM_2.5_ concentrations around their long-term city-specific trends were related to year-to-year variations in cause-specific admissions in each city. With this approach, we eliminated all confounding by covariates that varied across cities because this was a city-specific analysis, and by covariates whose long-term trends coincided with trends in PM_2.5_ within cities because those trends were removed. We assumed that year-to-year differences in PM_2.5_ concentrations around their city-specific trends were driven by year-to-year variations in the percent of time the city was downwind from more- or less-polluted areas and year-to-year variations in wind speed and inversions. Long-term changes in other exposures, such as changes in smoking rates and socioeconomic status, should be captured in the long-term trends, for which we adjusted. We think it is implausible that, for example, year-to-year variations in smoking rates around the long-term trend within a given city were correlated with year-to-year fluctuations in pollution concentrations driven by back trajectories or other such phenomena. Assuming this statement is true, our exposure variations were random with respect to other risk factors for admissions, and hence, our models should provide an unbiased estimate of the effects of PM_2.5_.

Moreover, we adjusted for any previous admission for CHF, COPD, MI, or diabetes and number of days spent in intensive and coronary care units. We also adjusted for ZIP-code level median income as a proxy for SES. All models were stratified by age (in 1-year intervals), sex, race (as white, black, and other), and year of follow-up.

City-specific effect estimates were pooled in a second stage, using a random effects meta-analysis (Berkey et al. 1998; Riley et al. 2011). Thus, in the “Results” section, we present the pooled estimates for each outcome as hazard ratios (HR) per 1-μg/m^3^ increase in PM_2.5_.

Further, we assessed potential effect modification by sex. In the city-specific models (first stage), we included an interaction term between PM_2.5_ concentrations and sex. We then pooled the city-specific coefficients of the interaction terms in a random effects meta-analysis and assessed whether the pooled effect estimate was significantly different from zero at the 0.05 level.

Finally, to assess whether the association between PM_2.5_ and neurological admissions was nonlinear, we repeated our main analysis using PM_2.5_ quartiles as a categorical variable.

For our statistical analyses, we used SAS software, version 9.3 (SAS Institute Inc., Cary, NC, USA), and R Statistical Software, version 2.14.1 (R Core Team 2014).

Sensitivity analyses. To assess the robustness of our findings, we conducted two sensitivity analyses, following the same methods as in the main analyses. First, given that one of the suggested biological pathways for the effects of PM_2.5_ on neurodegeneration is through inflammation (Block et al. 2007), adjusting for prior admissions for cardiovascular causes, that is, MI and CHF, might have meant that we adjusted for a proxy for a potential mediator (inflammation). To investigate this further, we repeated the analyses without adjusting for prior MI and CHF hospitalizations.

Moreover, because Medicare enrollees entered our cohort at the age of 65, there was no information on whether they had been hospitalized for any of the outcomes of interest at a younger age. To address this further, in an effort to remove potentially prevalent cases, we repeated our analyses, removing subjects who had been hospitalized for these outcomes during their first 2 years of follow-up and following the remaining participants from the third year of follow-up onward.

## Results

We included data from 50 cities in our analyses. The number of subjects and cause-specific admissions are presented in Table 1. Overall, our cohort consisted of approximately 9.8 million subjects, and in total, we observed 119,425 PD, 266,725 AD, and 203,463 dementia first admissions (either as primary or secondary causes). Across cities, the mean age in our cohort was 75.6 years (SD = 7.6); 57.3% of the subjects were female, and 80.4% were white. The average PM_2.5_ concentration was 12.0 μg/m^3^ (SD = 1.6, IQR = 3.8 μg/m^3^).

**Table 1:** Number of subjects, cause-specific admissions, and estimated hazard ratios for Parkinson’s disease, Alzheimer’s disease, and dementia.

Results	PD	AD	Dementia
Main analysis
Total population	9,817,806	9,817,806	9,817,806
Number of admissions	119,425	266,725	203,463
HR (95% CI) per 1 μg/m^3^	1.08 (1.04, 1.12)	1.15 (1.11, 1.19)	1.08 (1.05, 1.11)
HR (95% CI) per 5 μg/m^3^	1.44 (1.22, 1.70)	2.00 (1.70, 2.35)	1.46 (1.29, 1.66)
Excluding cases in the first 2 years after enrollment
Total population^*a*^	8,011,978	7,976,136	7,897,538
Number of admissions	80,788	202,614	143,888
HR (95% CI) per 1 μg/m^3^	1.07 (1.03, 1.11)	1.15 (1.10, 1.19)	1.07 (1.04, 1.11)
Abbreviations: AD, Alzheimer’s disease; HR, hazard ratio; PD, Parkinson’s disease. ^***a***^The number of total subjects for this sensitivity analysis is different by outcome, depending on the number of excluded cases in the first 2 years of follow-up by outcome.			

**Figure 2:**
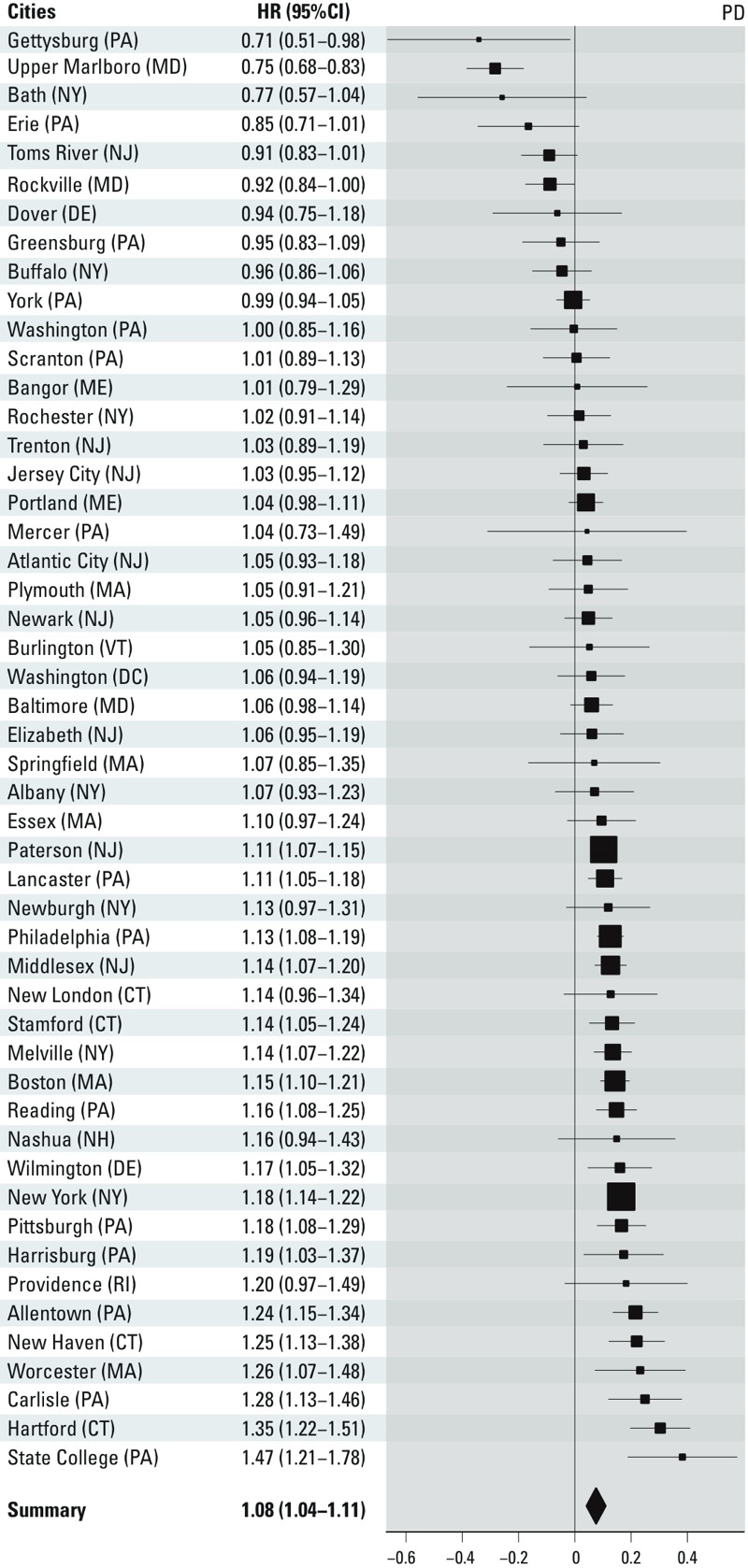
City-specific PM_2.5_ effect estimates on PD admissions, presented as log(HR) (95% CI) per 1-μg/m^3^ increase in PM_2.5_. PD, Parkinson’s disease. The size of the symbol used for the effect estimate is proportional to its precision.

**Figure 3:**
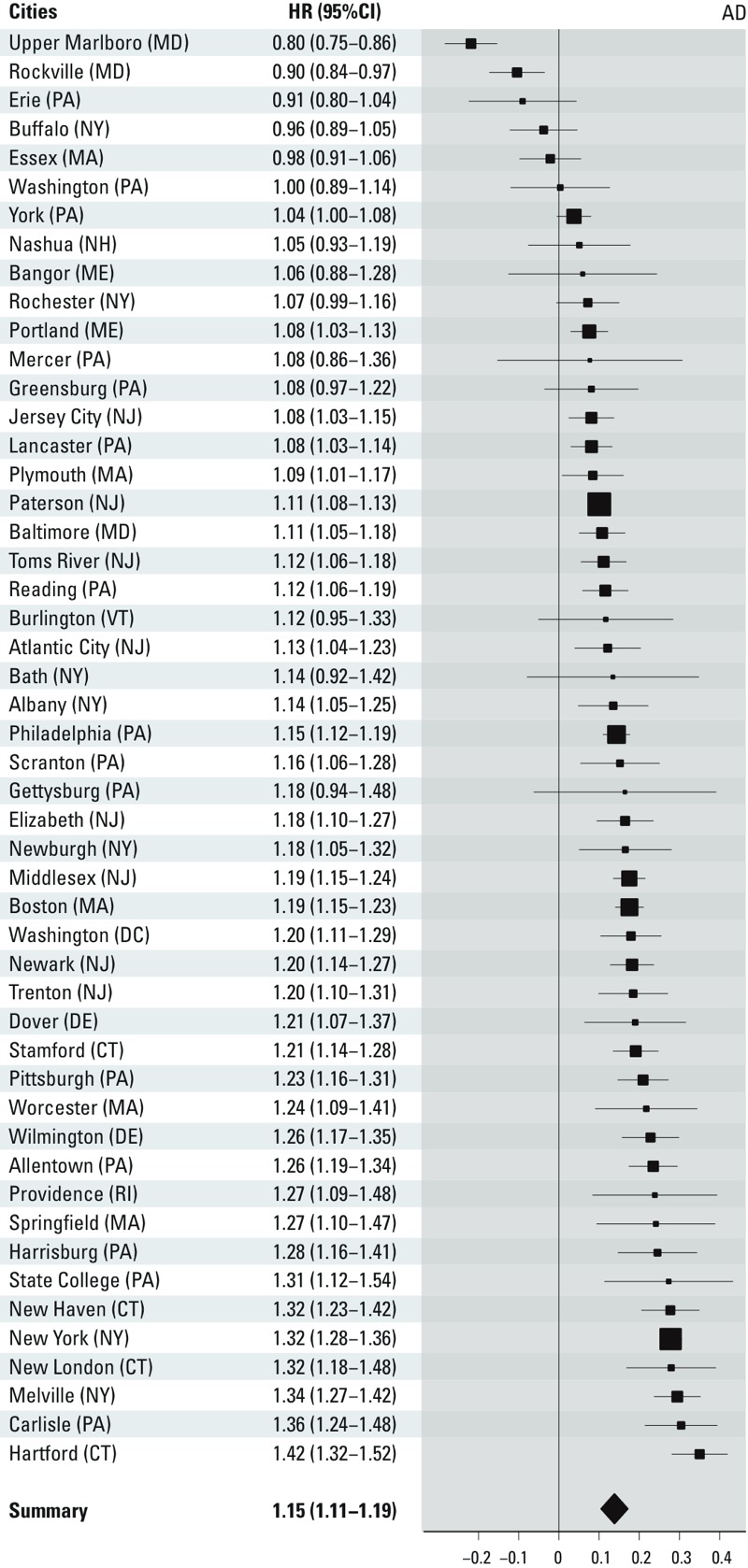
City-specific PM_2.5_ effect estimates on AD admissions, presented as log(HR) (95% CI) per 1-μg/m^3^ increase in PM_2.5_. AD, Alzheimer’s disease. The size of the symbol used for the effect estimate is proportional to its precision.

**Figure 4:**
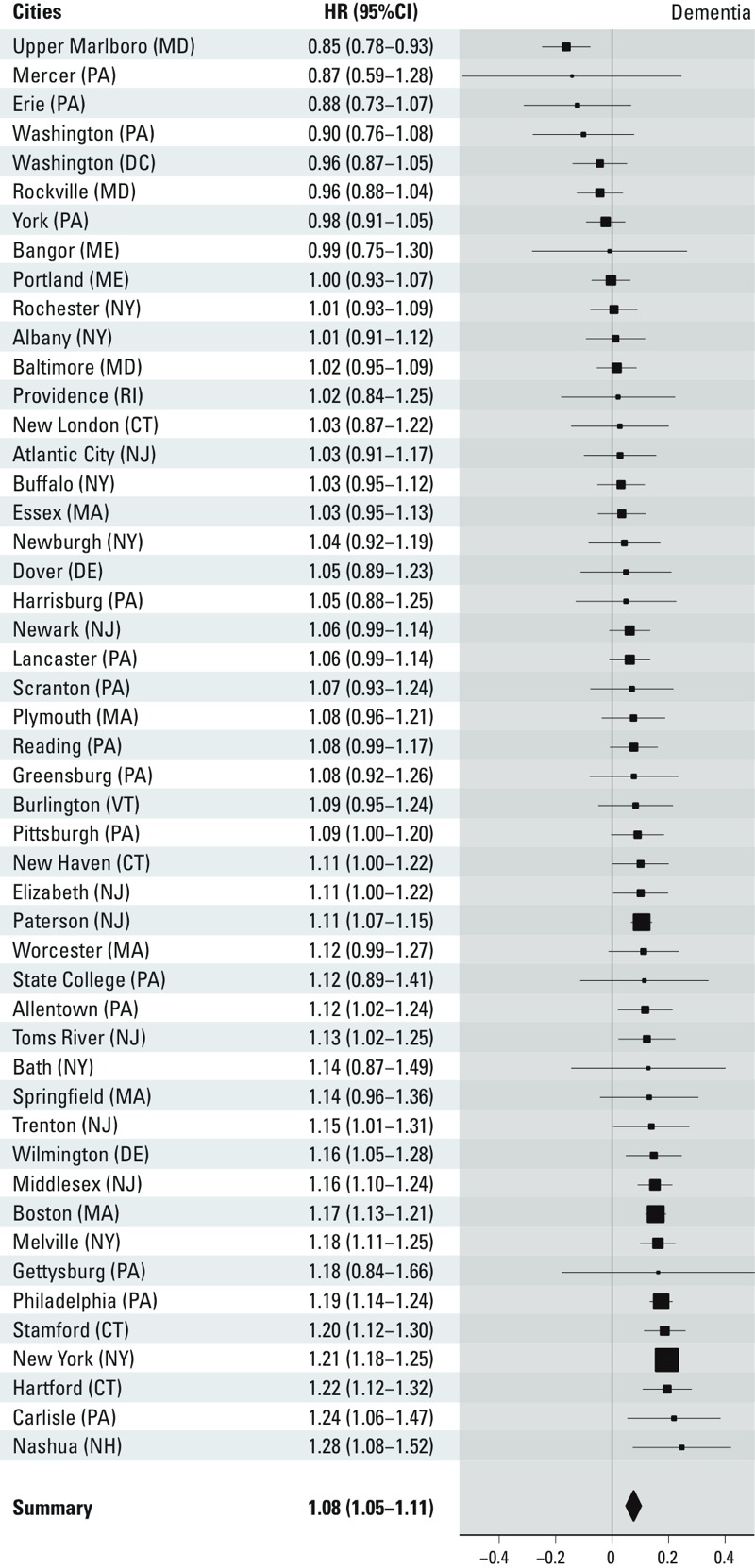
City-specific PM2.5 effect estimates on dementia admissions, presented as log(HR) (95% CI) per 1-μg/m3 increase in PM2.5. The size of the symbol used for the effect estimate is proportional to its precision.

City-specific estimates are presented in Figures 2–4. Overall, we observed statistically significant, positive pooled effect estimates of PM_2.5_ concentrations on all three outcomes of interest. Specifically, we observed the following: for PD admissions, HR = 1.08 (95% CI: 1.04, 1.12); for AD admissions, HR = 1.15 (95% CI: 1.11, 1.19); for dementia admissions, HR = 1.08 (95% CI: 1.05, 1.11) per 1-μg/m^3^ increase in annual PM_2.5_ city-wide exposure. We detected significant heterogeneity in the estimates across cities for all outcomes (*p* < 0.001).

For comparability with other long-term PM_2.5_ studies (e.g., Beelen et al. 2014) we also present our results per 5-μg/m^3^ increase (Table 1). We found no evidence of a non-linear relationship, as all observed associations by quartiles increased monotonically (results not shown).

We observed no statistically significant effect modification by sex for any outcome (all interaction *p* > 0.05) (data not shown). We found the largest by-sex difference across the estimated HRs for AD admissions, with HR = 1.16 (95% CI: 1.12, 1.21) for men and 1.14 (95% CI: 1.10, 1.18) for women (*p*-interaction = 0.58).

*Sensitivity analyses.* Our estimated HRs did not change when we repeated the analyses excluding any prior MI or CHF admission as variables from our first-stage model (results not shown).

The number of subjects and outcome-specific admissions when we excluded potentially prevalent cases are presented in Table 1. The estimated HRs in this sensitivity analysis were very similar to the HRs estimated in the main analysis.

## Discussion

We conducted a large-scale, multi-city study to estimate the impact of long-term PM_2.5_ city-wide exposure on city-wide hospital admissions for neurological outcomes, using data from Medicare enrollees in the northeastern United States. We followed approximately 9.8 million subjects from 1999 to 2010 and observed statistically significant, positive associations for all three outcomes of interest: first admission for PD, AD, and dementia. Our results were robust to the sensitivity analyses we conducted.

Although some authors have reported positive associations between PM_2.5_ exposure and reduced cognitive function (Gatto et al. 2014; Ranft et al. 2009), no epidemiologic studies have investigated the effects of long-term PM_2.5_ exposure on PD and AD. Recently, in an analysis of short-term PM_2.5_ effects, Zanobetti et al. (2014) reported a significant increase in PD-related hospitalizations after exposure to increased 2-day average PM_2.5_ levels. Only a few studies have examined the impact of long-term exposure to airborne metals on PD. Urban PM_2.5_ contains metals (Seinfeld and Pandis 2006), and the PM_2.5_ metal concentrations depend on the sources of PM_2.5_ in each city (Kioumourtzoglou et al. 2014a; Lall et al. 2011). Finkelstein and Jerrett (2007) observed increased odds ratios for a physician’s diagnosis of PD after exposure to particulate manganese. Similarly, using Medicare data, Willis et al. (2010) found increased incidence rates of PD among subjects living in counties with high reported industrial release of manganese or copper. Finally, Palacios et al. (2014) reported elevated, albeit statistically nonsignificant, associations between airborne mercury levels and PD in a cohort of elderly women.

Even though the direct epidemiologic evidence linking PM_2.5_ exposure to neurodegenerative diseases is sparse, toxicological studies have been published proposing several potential biological pathways (Block and Calderón-Garcidueñas 2009; Block et al. 2012). One potential pathway, for instance, is through oxidative stress: air pollution exposures have been repeatedly linked to oxidative stress (Chuang et al. 2007; Kim et al. 2004; Li et al. 2003; Sørensen et al. 2003). Furthermore, several studies reported evidence suggesting that oxidative stress plays a key pathogenic role in AD (Bonda et al. 2010; Huang et al. 2004; Su et al. 2008; Zhu et al. 2004). Inflammation has also been related to both air pollution exposure and neurodegeneration (Block and Calderón-Garcidueñas 2009). Both short- and long-term exposure to PM_2.5_ has been linked to increases in blood inflammatory markers (Dubowsky et al. 2006; Hoffmann et al. 2009). Inflammatory processes are thought to play an important role in the pathogenesis of both PD (McGeer and McGeer 2004) and AD (Wyss-Coray 2006).

Given the design of our study and the use of administrative data, we were not able to assess whether air pollution was associated with the onset of neurodegeneration. Rather, we assessed whether year-to-year fluctuations in PM_2.5_ concentration were associated with increases in hospital admissions for neurologic disorders. Thus, our findings indicate that air pollution likely accelerates the progression of neurodegeneration, potentially after the onset of disease.

The role of inflammation in the progression of neurodegeneration has been consistently reported (Cunningham et al. 2005; Teeling and Perry 2009). Cunningham et al. (2009) noted that inflammation primes the brain, making it more vulnerable to future inflammatory insults, which in turn change the rate of neurodegeneration and accelerate disease progression. Furthermore, exposure to increased PM_2.5_ levels in general, or to traffic particles in particular, have been associated with a series of intermediate outcomes, which in turn have been linked to more rapid cognitive decline or acceleration of AD progression. Examples of these intermediate outcomes include increased blood homocysteine (Oulhaj et al. 2010; Qiao et al. 2014; Ren et al. 2010), increased hypertension (Foraster et al. 2014; Goldstein et al. 2013; Li et al. 2011), narrower arteriolar diameters (Adar et al. 2010), and increased rates of ischemic stroke (Regan et al. 2006; Wellenius et al. 2012).

Our study has some limitations. First, outcome misclassification is a potential concern. We defined as our outcomes of interest the first hospital admission due to PD, AD, or dementia. Hospital admissions, however, might be recorded with misclassifications. A validation study of PD hospital discharges in Denmark, for instance, observed that approximately 82% of the reported PD admissions were accurate (Wermuth et al. 2012). We would expect any resulting bias, however, to be toward the null.

Exposure measurement error is also likely and, if present, has also been shown to bias results towards the null (Kioumourtzoglou et al. 2014b). Furthermore, it is likely that mobility and/or memory issues during the early stages of these conditions might decrease the amount of time spent outdoors, which could further bias the effect estimates towards the null. Nonetheless, given the average age of Medicare enrollees, mobility issues among non-cases are also likely (Kannus et al. 1996; Melton 1996).

Additionally, Medicare is an open cohort into which subjects enter when they turn 65 years old. Given no prior information on their health status, some subjects could have been hospitalized for the outcome of interest before turning 65. To examine whether their inclusion in our analyses affected our estimates, we conducted a sensitivity analysis excluding potentially prevalent cases, which indicated that our results were robust.

We detected significant effect heterogeneity in the estimates across cities for all outcomes. This finding could be partially attributed to the large number of cities and participants in our study, which provided ample power to detect heterogeneity even across the smallest differences in estimates. It is also likely that other factors contributed to this heterogeneity. For example, particle composition has been shown to modify the association between long-term exposure to air pollution and other outcomes, such as mortality (Kioumourtzoglou et al. 2015). Nevertheless, it should be noted that the majority of the estimates across cities were positive and many of those were significantly so (Figures 2–4), indicating that this heterogeneity only reflected differences across harmful estimates.

Finally, although residual confounding cannot be excluded, it is not likely to have occurred in our study. Individual-level potential confounders, such as smoking and other lifestyle factors, are not available for Medicare enrollees, as these data are collected largely for utilization and cost statistics and not for epidemiological analyses. We did, however, select a study design that did not allow potential confounders that varied across cities, or long-term trends, to affect our estimates. Moreover, we adjusted for age, race, sex, and SES, as well as for any prior cardiopulmonary admission and severity of disease. In addition, chronic PM_2.5_-mortality studies using Medicare data have yielded very similar results to studies that adjusted for more individual-level confounders (Eftim et al. 2008; Zeger et al. 2008).

To our knowledge, this has been the first large-scale, multi-site epidemiologic study to examine the association between air pollution and hospital admissions due to the most common neurodegenerative diseases. We observed statistically significant, positive associations between long-term PM_2.5_ city-wide exposures and PD, AD, and dementia, supporting our hypothesis. In light of our limitations, our results should be viewed as preliminary; our findings provide the basis for further exploration in large epidemiologic studies with validated outcomes and more detailed information on potential individual-level confounders. Such studies are of crucial importance, as the implications for public health are tremendous, especially given the anticipated increase in life expectancy.
